# Color and time perception: Evidence for temporal overestimation of blue stimuli

**DOI:** 10.1038/s41598-018-19892-z

**Published:** 2018-01-26

**Authors:** S. Thönes, C. von Castell, J. Iflinger, D. Oberfeld

**Affiliations:** 1 Experimental Psychology, Institute of Psychology, Johannes Gutenberg-Universität Mainz, Mainz, Germany; 2 Leibniz Research Centre for Working Environment and Human Factors, TU Dortmund, Dortmund, Germany

## Abstract

The perceived duration of a visual stimulus depends on various features, such as its size, shape, and movement. Potential effects of stimulus color have not been investigated in sufficient detail yet, but the well-known effects of arousal on time perception suggest that arousing hues, such as red, might induce an overestimation of duration. By means of a two-interval duration discrimination task in the sub-second range, we investigated whether participants overestimate the duration of red stimuli in comparison to blue stimuli, while controlling for differences in brightness (individual adjustments by means of flicker photometry) and saturation (colorimetric adjustment in terms of the CIELAB color space). Surprisingly, our results show an overestimation of the duration of blue compared to red stimuli (indicated by a shift of the point of subjective equality), even though the red stimuli were rated as being more arousing. The precision (variability) of duration judgments, i.e., the duration difference limen, did not differ between red and blue stimuli, questioning an explanation in terms of attentional processes.

## Introduction

An accurate perception of time and the ability to precisely time the duration of temporal intervals are essential conditions for appropriate behavioral control. Over the last decades, several different issues concerning time perception have been investigated, such as whether there is a dedicated mechanism for judging duration (“internal clock”), and which internal and external factors are relevant for our perception of time^1–4^. In the present study, we investigated whether the perceived duration of a visual stimulus is affected by its color.

A potential effect of color on time perception arises because of effects of color on emotion. It is well-established that red light, which consists predominantly of longer wavelengths, induces higher levels of arousal^5–7^. Beside the hue of a color stimulus (e.g., green, red, or blue), it is, however, important to consider the two other perceptual dimensions of color: brightness (perceived intensity of the light; e.g., bright vs. dark) and saturation (difference to an achromatic stimulus, i.e., a neutral grey or white)^8^. A red hue causes higher arousal than a blue hue for colors of equal brightness and saturation, at least when the colors have a medium to high level of saturation^9^. In research on time perception, especially in the context of duration judgments, arousal represents an important, sometimes even the main driving factor. The relationship between arousal and duration judgments can be described in terms of the prominent pacemaker-accumulator models of time perception^10–12^, also termed internal-clock models. These models propose a clock device consisting of two subcomponents: a *pacemaker* emitting pulses and an *accumulator* counting these pulses. The amount of pulses accumulated during a defined temporal interval (e.g., during the presentation of a visual stimulus) is positively correlated with the perceived length of this interval. Arousal is assumed to directly influence the rate of pulse emission by the pacemaker^11,13^. Higher levels of arousal are associated with an increased rate of pulse emission. Imagine that a red and a blue stimulus are both presented for 500 ms, and that the red stimulus causes higher arousal than the blue stimulus. Then the number of clock pulses accumulated during the 500 ms stimulus duration will be larger for the red than for the blue stimulus due to the arousal-induced increase in pulse rate. According to the internal-clock model, this should result in a longer perceived duration of the red compared to the blue stimulus. Such effects of arousal on time perception were reported in several experiments manipulating the level of arousal^13–15^, for example by means of emotional valence of presented facial expressions^16–18^, threatening character^19^, and erotic content^20^.

As higher levels of saturation and brightness also induce more arousal (irrespective of a specific hue)^9^, it is therefore important to carefully control for these non-hue-related aspects of color stimuli. While brightness and saturation may also influence our perception of duration (via arousal), we focused on effects of hue in the present study as this dimension is most prominent in the everyday experience of color. Effects of hue on time perception have not been studied extensively, and the existing studies often failed to control for differences in brightness and saturation. Moreover, it is preferable to realize isoluminance (brightness matching) of different color stimuli on the individual level, for example by means of flicker photometry^8^, which was not done in all previous studies.

For instance, Gorn, *et al*.^21^ reported that the duration of a red screen was overestimated in comparison to a blue screen. In this particular study, hue, brightness, and saturation were manipulated independently. However, the dependent measures (quickness ratings of download speed) are rather uninformative, and the brightness was matched according to colorimetric measurements and not adjusted individually. A small but significant temporal overestimation of red stimuli compared to blue stimuli, restricted to male participants, was found by Shibasaki and Masataka^22^, using a one-interval duration-discrimination task (temporal bisection task)^3^. However, brightness and saturation were not controlled. Contrary to the reports of temporal overestimation of red stimuli, an opposite effect was found by Smets^23^: In a verbal estimation task and a time reproduction task (for a description of these tasks see a recent review by Thönes and Oberfeld^24^), participants overestimated the duration of blue stimuli in comparison to red stimuli. While brightness was matched individually, the study by Smets failed to control for possible differences in the saturation of the different color stimuli. Further studies on effects of color on time perception did also not provide clear evidence for a temporal overestimation of red stimuli^25,26^, and may even challenge the notion of red being more arousing than blue^27,28^.

On a more general level, the performance of participants in time perception tasks can be characterized in terms of *accuracy*, which indexes the (signed) deviation of a judgment from the veridical value, and in terms of *precision*, which refers to the *variability* of the judgments^24^. Most of the previous studies considered only effects of color on the accuracy of time perception, and ignored potential effects on precision.

To overcome these limitations, the present study investigates potential effects of hue on time perception by means of a two-interval duration-discrimination task. The presented red and blue stimuli were individually brightness matched and the saturation was controlled colorimetrically. The data were analyzed both in terms of accuracy and precision. Based on the aforementioned pacemaker-accumulator models of interval timing^11^, we assumed that participants overestimate the duration of stimuli of red hue (high arousal) in comparison to stimuli of blue hue (low arousal). In the two-interval duration-discrimination task, two stimuli of slightly different duration are presented successively, and the participant has to indicate which stimulus was longer in duration. Non-temporal stimulus properties, such as the hue, may result in a longer or shorter perceived duration of stimuli with identical physical duration. A systematic difference in perceived duration between, e.g., a red and a blue stimulus is indexed by a deviation of the point of subjective equality (PSE) from the point of objective equality. If we denote the duration of the first stimulus by *T*_1_ and the duration of the second stimulus by *T*_2_, then the PSE is defined as the difference in duration (*T*_2_ − *T*_1_) at which the participants are indifferent as to which of the two stimuli has a longer duration. Thus, the PSE is the 50% point on the psychometric function (PMF) relating the difference in duration between second and first stimulus (*T*_2_ − *T*_1_) to the proportion of trials on which a participant responded that the duration of the second stimulus was longer, which we denote by *P*(“2^nd^ stimulus longer”). If the PSE differs between the two orders of presentation (red-blue versus blue-red), this indicates a relative over- or underestimation of duration depending on the hue, and thus an effect of hue on the *accuracy* of time perception.

In addition, data from two-interval duration-discrimination tasks provide information about the *precision* of temporal judgments in terms of the duration difference limen (DL) that can be estimated from the psychometric function. While it has been shown repeatedly that increased levels of arousal affect the accuracy of temporal judgments, leading to an overestimation of duration, arousal did not affect the precision of temporal judgments in previous studies^18,20,29^. Therefore, since arousal was assumed to be the main driving factor for effects of hue on time perception, we expected an overestimation of red stimuli in comparison to blue stimuli, but no effect of hue on the precision of temporal judgments, measured by the duration DL.

## Results

### Duration judgments

On each trial, the participants compared the duration of two sequentially presented visual stimuli that were each either red or blue. One of the two stimuli (standard; *s*) always had a duration of 500 ms, and the duration of the other stimulus (comparison; *c*) was varied from 300 to 700 ms in steps of 50 ms. The comparison was presented first (stimulus order <*cs*>) or second (stimulus order <*sc*>) with equal probability. In a first step, we analyzed the proportion of “second stimulus longer”-responses, *P*(“2^nd^ stimulus longer”), as a function of color sequence on trials where the durations of the first and the second stimulus were identical (500 ms). Note that the color sequences blue-red (b-r) and red-blue (r-b) provide the data for the relevant between-color comparisons. In b-r trials, “second stimulus longer”-responses represent “red longer”-responses. In r-b trials, “second stimulus longer”-responses represent “blue longer”-responses. We also investigated potential differences between the same-color conditions blue-blue (b-b) and red-red (r-r). An absence of such effects would strengthen the results obtained for the between-color comparisons.

**Figure 1:**
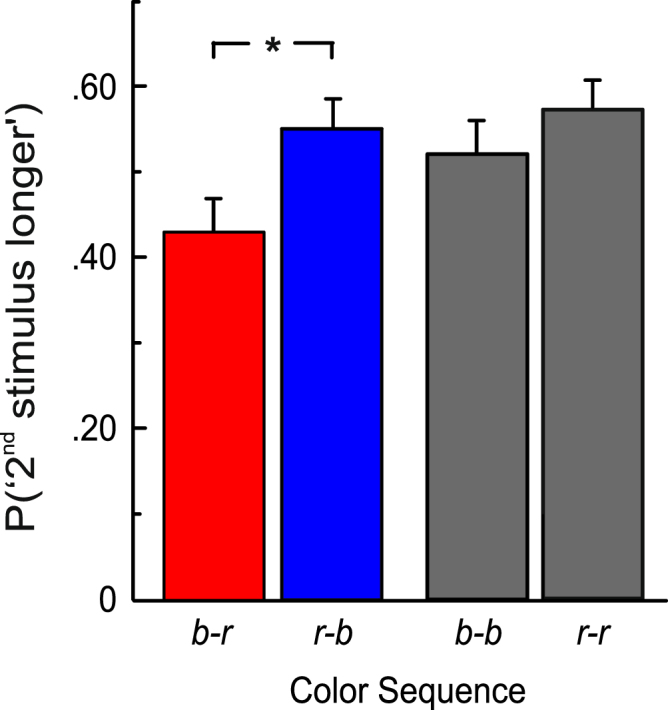
Mean proportion of “Second stimulus longer”-responses (*P*(“2^nd^ stimulus longer”)) on trials where the duration of both stimuli was 500 ms, as a function of color sequence. Error bars indicate +1 standard error of the mean (SEM). *N* = 13. *Indicates statistically significant differences (*p* < 0.05).

As presented in Fig. 1, when the duration of both stimuli was identical (500 ms), the mean *P*(“2^nd^ stimulus longer”) was larger in trials with a blue stimulus at the second temporal position relative to trials with a red stimulus at the second position. We analyzed the effect of stimulus color on *P*(“2^nd^ stimulus longer”) by means of a repeated-measures analysis of variance (rmANOVA) with the within-subjects factor color sequence (b-r, r-b, b-b, r-r). We used a univariate approach with Huynh-Feldt correction for the degrees of freedom^30^. The correction factor $\mathop{\varepsilon }\limits^{ \sim }$ is reported, and partial η^2^ is reported as a measure of association strength. By means of post-hoc paired-samples *t*-tests (two-tailed), we compared the between-color conditions b-r and r-b and the same-color conditions b-b and r-r. An α-level of 0.05 was used for all analyses. For the pairwise comparisons, we report Cohen’s *d*_*z*_^31^ as a measure of effect size in a within-subjects design. Because non-normally distributed measures can cause problems in repeated-measures ANOVAs^32^, the proportions were arcsin-square-root transformed^33^ to obtain a closer approximation to the normal distribution. In the rmANOVA, the effect of color sequence was significant, *F*(3, 36) = 5.768, $\mathop{\varepsilon }\limits^{ \sim }$ = 1.0*, p* = 0.003, η_p_^2^ = 0.325. Post-hoc paired-samples *t*-tests showed a significant difference in *P*(“2^nd^ stimulus longer”) between the color sequences b-r and r-b, *t*(12) = 2.503, *p* = 0.028, *d*_*z*_ = 0.69. Thus, when the duration of the two stimuli was identical, blue stimuli were more frequently judged as longer than red stimuli than vice versa, indicating a relative overestimation of the duration of the blue stimuli. The difference between the same-color conditions (b-b and r-r) did not reach statistical significance, *t*(12) = 2.161, *p* = 0.052.

**Figure 2:**
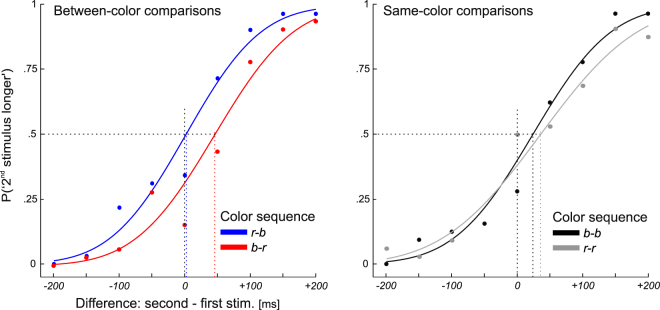
Example psychometric functions for one participant. Left panel: color sequence r-b (blue line) compared to b-r (red line). The line colors represent the hue of the second stimulus. Right panel: color sequence b-b (black line) compared to r-r (gray line). The vertical lines represent the point of objective equality and the points of subjective equality (PSEs). Each psychometric function is based on 288 trials.

In the second step, we fitted a cumulative-normal psychometric function (PMF) to the observed responses and determined the point of subjective equality (PSE) and the duration difference limen (DL) for each of the 13 participants, for each color sequence (b-r, r-b, b-b, r-r). For analyses including the factor comparison position, please refer to the Supplementary Information. The PMF plots *P*(“2^nd^ stimulus longer”) as a function of the duration difference between the second and the first stimulus. The DL was defined as half the difference between the 75%- and the 25%-point on the PMF. Example PMFs for one subject are shown in Fig. 2, for the two between-color comparisons (color sequences b-r and r-b; left panel) and the two same-color comparisons (color sequences b-b and r-r; right panel). In the example, the PMFs in the left panel show that, for the color sequence r-b, the participants perceived the second stimulus to be identical in duration to the first stimulus when the actual duration difference was close to 0 ms. In contrast, for the color sequence b-r, the two stimuli were perceived as identical in duration when the second stimulus (red) was 45 ms longer than the first stimulus (blue). This shift of the psychometric function to the right, that is, a larger PSE than for the color sequence r-b, indicates that the duration of the blue stimulus was overestimated relative to the duration of the red stimulus. Note also that a steeper slope of the PMF (i.e., a smaller DL) indicates higher temporal discrimination sensitivity (precision).

**Figure 3:**
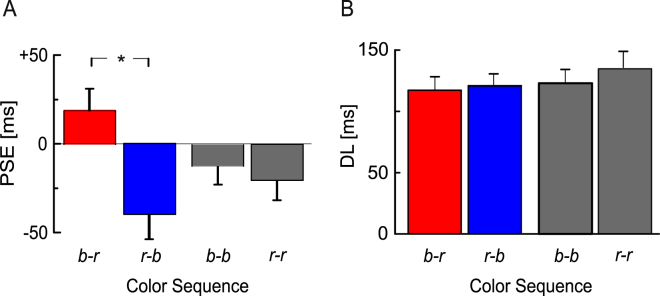
Mean point of subjective equality (PSE; panel A) and mean difference limen (DL; panel B) as a function of color sequence. Error bars indicate +/−1 standard error of the mean. *N* = 13. *Indicates statistically significant differences (*p* < 0.05).

As presented in the left panel of Fig. 3, the mean PSEs were smaller when the second stimulus was blue compared to when the second stimulus was red, indicating a relative overestimation of the duration of the blue stimuli.

The data were analyzed by means of two rmANOVAs including the within-subjects factor color sequence (b-r, r-b, b-b, r-r). In the first ANOVA, we analyzed the effect of color sequence on the PSE. The effect of color sequence was significant, *F*(3, 36) = 6.857, $\mathop{\varepsilon }\limits^{ \sim }$ = 0.580, *p* = 0.007, η_p_^2^ = 0.364. A post-hoc paired-samples *t*-test comparing the between-color conditions b-r and r-b showed that the PSEs were significantly smaller when the second stimulus was blue rather than when it was red, *t*(12) = 2.917, *p* = 0.013, *d*_*z*_ = 0.81, confirming an overestimation of the duration of blue stimuli relative to red stimuli. A post-hoc *t*-test comparing the color sequences b-b and r-r did not indicate a significant difference between the PSEs observed for the two same-color sequences, *t*(12) = 1.645, *p* = 0.126.

Note also that the slightly negative PSEs for the same-color sequences (b-b and r-r) indicate a time-order-error in the sense that the two stimuli were perceived as identical in duration when the second stimulus was actually slightly shorter than the first.

In the second ANOVA, we analyzed the effect of color sequence on the DL. There was no significant effect of color sequence on the DL, *F*(3, 36) = 1.957, $\mathop{\varepsilon }\limits^{ \sim }$ = 0.725, *p* = 0.158, η_p_^2^ = 0.140. As seen in panel B of Fig. 3, the DLs were relatively similar for the four color sequences, except for a slightly higher DL for sequence r-r. In an additional analysis presented in the Supplementary Information, we estimated separate DLs for trials on which the comparison was presented in the first interval and trials on which the comparison was presented in the second interval^34^. This analysis showed no significant effect of color sequence on the DLs, compatible with the analysis reported above. The interaction between color sequence and position of the comparison was also not significant. However, the DLs were significantly larger on trials where the CI was presented first, compatible with previous studies^34–36^.

### SAM ratings

**Figure 4:**
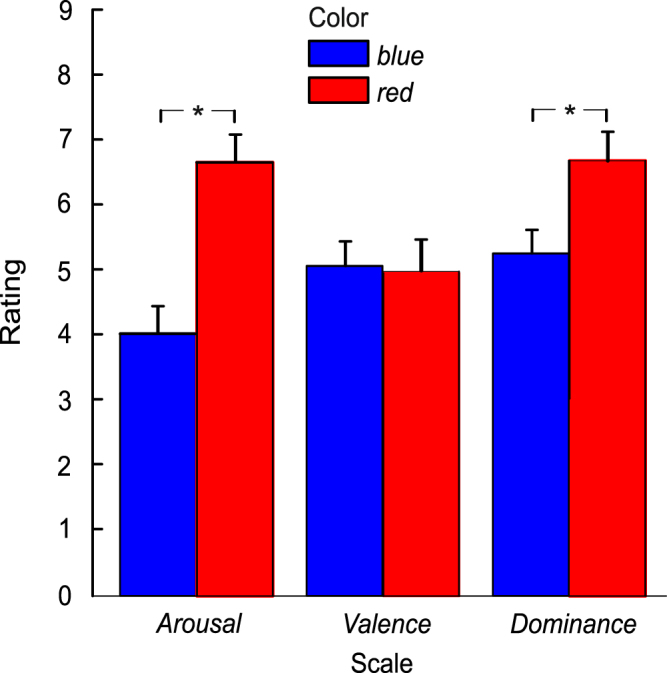
Mean SAM ratings on each scale (arousal, valence, and dominance) as a function of stimulus color. Error bars indicate +1 standard error of the mean. *N* = 13. *Indicates statistically significant differences (*p* < 0.05).

In each of the four experimental sessions, the emotional responses to the blue and red stimulus were assessed by means of the nonverbal self-assessment manikin (SAM) scales^37^. One rating was obtained before the duration discrimination task, and one rating after the task. The mean arousal, valence, and dominance ratings for the red and blue stimulus are depicted in Fig. 4, averaged across sessions (1 to 4) and across the pre- and post-task ratings obtained in each session. The data indicate that the red stimulus was rated as more arousing and more dominant in comparison to the blue stimulus, whereas the valence ratings were approximately equal for the two colors.

The rating data were analyzed by means of three separate paired-samples *t*-tests for arousal, valence, and dominance. The independent variable was stimulus color (red versus blue). Stimulus color had a significant effect on arousal, *t*(12) = 3.996, *p* = 0.002, *d*_*z*_ = 1.11, and dominance, *t*(12) = 2.511, *p* = 0.027, *d*_*z*_ = 0.70. There was no significant effect of color on valence, *t*(12) = 0.084, *p* = 0.937.

Additionally, by means of correlation analyses, we investigated whether the difference in the PSE between the color sequences red-blue and blue-red was correlated with the difference in arousal ratings between the red and the blue stimulus. This was not the case, *r* = 0.199, *p* = 0.515.

## Discussion

In the present study, we investigated whether the perceived duration of a visual stimulus is affected by its color. Based on the well-known effects of arousal on time perception and the arousing character of red hues, we assumed that participants should overestimate the duration of a red stimulus in comparison to a blue stimulus. By means of a two-interval duration-discrimination task, we obtained independent measures of temporal accuracy (PSEs) and temporal precision (DLs). Unexpectedly, our data clearly indicate a temporal overestimation of the blue stimulus in comparison to the red stimulus. Based on the analyses of the psychometric functions, blue and red stimuli were perceived to be of equal duration when the blue stimulus was in fact 60 ms (12%) shorter than the red stimulus (mean difference in PSEs). This effect was large in size according to the classifications by Cohen^31^. This result is particularly surprising because the red stimulus induced significantly higher levels of arousal according to the emotion ratings collected in the experiment, as expected. However, the difference in the PSEs between red and blue stimuli was not correlated with the difference in arousal ratings between red and blue, thus questioning arousal as the main driving factor in the context of our study.

It is a general uncertainty in research on emotion whether, for example, the arousal rating for an “emotional” picture reflects the actual emotional state of the observer while viewing the picture, or rather a “semantic” judgment of the content of the picture. For instance, as noted by a reviewer, a demand characteristic in the sense of ‘everyone thinks red is more arousing than blue’ could have contributed to the arousal ratings. On the other hand, it is not impossible that knowing that ‘everyone thinks red is more arousing than blue’ causes a state of higher arousal, broadly in the sense of a self-fulfilling prophecy. Several studies reported a correlation between arousal ratings and changes in physiological markers of arousal (such as the skin conductance response), both for colors^9^ and emotional pictures^38^. This suggests that, e.g., the higher arousal ratings for red compared to blue stimuli are note merely a “cold” semantic judgment but in fact represent changes in the arousal state of the observers. This notwithstanding, even if we assumed the arousal ratings collected in our experiment to be “cognitive” rather than “emotional” judgments, and thus conclude that the stimulus color did not alter the arousal state of the participants, this does not explain the observed overestimation of the duration of blue stimuli.

Can the effect be explained in terms of attention? If specific attentional processes were underlying the effect, one might expect differences not only in the accuracy but also in the precision of the duration judgments. Increased selective attention in timing tasks has been shown to be associated with higher temporal precision^29^. Our results, however, do not indicate such an effect on the precision (DL) of temporal judgments, thus questioning an explanation in terms of attentional processes. Moreover, the time intervals of our duration-discrimination task were in the sub-second range, where attentional (cognitive) processes in time perception are typically assumed to be less relevant than at longer durations.

If one considers the earliest stage of visual perception, red and blue light of course results in a different activation pattern in human photoreceptors. The activation of S-cones relative to the activation of M- and L-cones will be higher under blue than under red light. However, we are not aware of physiological models suggesting a differential effect of different cone types on time perception. Red and blue light will also have different effects on the so-called non-visual photoreceptors. These melatonin-suppressing retinal ganglion cells^39–41^ project to the suprachiasmatic nuclei, which are involved in the regulation of circadian transitions between sleep and wakefulness^42,43^. The dominant peak in the wavelength spectrum of the blue primary of our CRT display (453 nm) is close to the peak sensitivity in the action spectrum of the Melanopsin-expressing ganglion cells at approximately 460 nm^40,44^. In contrast, the dominant spectral peak for the red primary was located at 625 nm, which can be considered to be outside the action spectrum of the Melanopsin-expressing ganglion cells. Thus, the blue stimuli could have caused a higher activation in the circadian system, and this system has been suggested to play a role for the perception of short durations^45,46^. However, due to the relatively low light intensity and the short presentation duration, it is not immediately clear whether significant effects in the circadian system can be expected for our stimuli. Also, the activation of the visual photoreceptors (rods and cones) modulates the activation of the Melanopsin-expressing ganglion cells^47^, which makes the prediction of the responses of the latter cells to colors of short and long wavelength more complicated. Thus, additional research would be required to identify a potential role of the non-visual photoreceptors for time perception.

In conclusion, our results show that the perceived duration of a stimulus is affected by its hue. Blue stimuli were temporally overestimated in comparison to red stimuli. This result is particularly surprising as the red stimuli were rated as being more arousing. While, in general, arousal is known to cause an acceleration of the internal clock, leading to an overestimation of duration, arousal is obviously not the main driving factor in the context of our experiment. Moreover, as the precision (variability) of duration judgments did not differ between red and blue stimuli, an alternative explanation for the effect of hue in terms of attentional processes remains also questionable. Thus, it remains for future research to identify the processes underlying the effects of color on time perception. Notwithstanding the discussed limitations of our study and the need for additional research, this line of research might contribute to the progression and modification of models of time perception and temporal processing.

## Method

### Participants

16 students (10 female) participated in the experiment in return for partial course credit. All participants were healthy and had normal or corrected-to-normal visual acuity. A test of color deficiency^48^ confirmed normal color vision in all participants (test plates 1, 4, 7, 13, 15, and 20 were presented under daylight conditions). Data from three participants were excluded from the analysis due to extremely poor performance in the duration discrimination task (data from one participant did not allow fitting the psychometric model; in two other cases, the difference limens of specific conditions were extremely large, 3161 and 14397 ms, respectively). The remaining 13 participants (10 female) ranged in age from 20 to 26 years (*M* = 21.77 y, *SD* = 1.96 y). The experiment was conducted according to the principles expressed in the Declaration of Helsinki. The study was approved by the Institutional Review Board (IRB) of the Institute of Psychology at the Johannes Gutenberg-Universität. Prior to the study, the IRB had informed us that in accordance with the department’s ethics guidelines no explicit ethics vote of the IRB was necessary for our study, because we tested only healthy adult volunteers, only harmless visual stimuli were presented, the experiment did not cause physical or psychological stress, no physiological parameters were measured, no sensitive data like personality or clinical scales were collected, and no misleading or wrong information was given to the participants. All participants participated voluntarily after having provided informed written consent. They were uninformed about the experimental hypotheses.

### Apparatus

The experiment was carried out in a soundproof booth with dimmed lights. Instructions and stimuli were presented by a computer equipped with a dual core E5700 processor and an Nvidia Quadro FX1400 graphics card. The screen was a NEC Model Multi Sync 90 F 19″ CRT display with a resolution of 1280 × 1024 pixels at a display rate of 89 Hz and a color depth of 32 bit. The participant’s head was steadied by a chin rest at a viewing distance of 85 cm from the screen. The stimuli were presented using Python 2.7. All responses were given by using a numeric keypad and a response box with two buttons.

### Stimuli and procedure

In a two-interval duration-discrimination task, on each trial, two color stimuli (disks with a diameter of 10° visual angle at the viewing distance of 85 cm) were presented successively in the center of the screen. The blank inter-stimulus interval (ISI) was 900 ms. On each trial, one stimulus was presented for 500 ms (standard interval; SI), while the duration of the other stimulus varied between 300 and 700 ms in steps of 50 ms (comparison interval; CI). The CI was presented in the first or second temporal position with equal probability. Four different sequences of the hues of the two stimuli were presented (red-blue [r-b], blue-red [b-r], blue-blue [b-b], red-red [r-r]). After the presentation of the second stimulus, the participant indicated which stimulus had been presented for a longer duration, using the two designated response buttons. No feedback was provided. After an inter-trial interval (ITI) of 3000 ms, the next trial started.

The blue stimulus was presented with the blue primary set to its maximum level (RGB = [0, 0, 1]). Its colorimetric values are shown in Table 1. In order to ensure isoluminance of the two color stimuli, the intensity of the red stimulus was individually adjusted at the beginning of session 1. Using the method of flicker photometry^8^, three heterochromatic brightness matches between blue and red were obtained per participant. The digital level of the red primary was varied, and the digital levels of the green and blue primary were set to 0. The participants were asked to increase or decrease the brightness of the red stimulus using the two response buttons until the subjective amount of flicker was minimal. The flicker frequency was 10 Hz.

**Table 1:** Colorimetric values of the blue and the red disk color. Columns *X*, *Y*, and *Z* display the CIE XYZ tristimulus values according to the 10° CIE 1964 standard observer^49^, columns L* and h* display the lightness and hue values according to the CIE LCh 1976 system^50^, column *S* displays the saturation values calculated from the LCh 1976 chroma (C*) values: *S* = C*^2^/(C*^2^ + L*^2^)^1/2^ · 100%^51^. L*, *S*, and h* are specified relative to a D65 white point. For the red stimulus, all values were averaged across the 13 remaining participants (*SD* in parentheses).

Disk color	*X*	*Y* (cd/m^2^)	*Z*	L*	*S*	h* (deg)
Blue	3.09	2.15	15.05	16.26	95.53	293.15
Red	3.65 (0.76)	2.07 (0.42)	0.48 (0.06)	15.72 (2.23)	92.21 (0.61)	32.46 (2.06)

In a within-subjects design, the three experimental factors (color sequence, CI position, and CI duration) were fully crossed. Each of the 4 (color sequence) × 2 (CI position) × 9 (CI duration) combinations was presented 16 times, resulting in a total of 1152 experimental trials per participant. Participants were tested in four experimental sessions, each lasting approximately 40 minutes. In each session, 288 trials of the duration-discrimination task were presented in random order. In session 1, participants received a short practice block (20 trials, randomly selected). In all sessions, the brightness of the red stimulus in the duration discrimination task was based on the individual brightness matches obtained in the first session.

Before the beginning of the duration-discrimination task, in each session, the emotional responses to the blue and red stimulus were assessed by means of the nonverbal self-assessment manikin (SAM) scales^37^. The emotional dimensions valence, arousal, and dominance are illustrated by nine horizontally arranged pictograms each. From left to right, for the valence dimension, the scale ranges from a smiling, happy figure to a frowning, unhappy figure. For the arousal dimension, it ranges from an excited, wide-eyed figure to a relaxed, sleepy figure. For the dominance dimension, it ranges from a tiny to a large figure. A computer-based version of the scales was used. The scales were displayed with numerical labels ranging from “1” to “9”, respectively. For the arousal scale, “1” corresponded to “calm” and “9” to “aroused”. For the valence scale, “1” and “9” corresponded to “unpleasant” and “pleasant”, respectively. For the dominance scale, “1” and “9” corresponded to “submissive” and “dominant”, respectively. The nine numbers were vertically aligned with the centers of the nine pictograms. For each of the three scales, participants rated their emotional state by entering one of the integer numbers 1 to 9 on the numerical keypad. First, the blue stimulus was rated in terms of valence, arousal, and dominance. Subsequently, the red stimulus was rated accordingly. The SAM ratings were used as a manipulation check, in order to test whether the red stimulus induced more arousal than the blue stimulus. After the duration-discrimination task, in order to increase the reliability of the SAM-ratings, a second set of ratings for the two color stimuli was collected in each session.

### Data availability

The datasets generated and analyzed during the current study are available from the corresponding author on reasonable request.

## Electronic supplementary material

Supplementary Information

## References

[1] Ivry RB, Spencer RMC (2004). The neural representation of time. Curr Opin Neurobiol.

[2] Wittmann M, van Wassenhove V (2009). The experience of time: neural mechanisms and the interplay of emotion, cognition and embodiment. Philosophical Transactions of the Royal Society B-Biological Sciences.

[3] Grondin S (2010). Timing and time perception: A review of recent behavioral and neuroscience findings and theoretical directions. Atten Percept Psycho.

[4] Matthews W, Meck W (2016). Temporal Cognition: Connecting Subjective Time to Perception, Attention, and Memory. Psychological Bulletin.

[5] Wilson GD (1966). Arousal Properties of Red Versus Green. Percept Motor Skill.

[6] Walters J, Apter MJ, Svebak S (1982). Color preference, arousal, and the theory of psychological reversals. Motivation and Emotion.

[7] Jacobs KW, Hustmyer FE (1974). Effects of Four Psychological Primary Colors on GSR, Heart-Rate and Respiration Rate. Percept Motor Skill.

[8] Wyszecki, G. & Stiles, W. S. *Color science: Concepts and methods, quantitative data, and formulae*. Wiley classics library edn, (John Wiley & Sons, 2000).

[9] Wilms, L. & Oberfeld, D. Color and emotion: effects of hue, saturation, and brightness. *Psychological Research* (2017). 10.1007/s00426-017-0880-8 28612080

[10] Gibbon J, Church RM, Meck WH (1984). Scalar timing in memory. Ann Ny Acad Sci.

[11] Treisman M (1963). Temporal discrimination and the indifference interval: Implications for a model of the “internal clock”. Psychological Monographs.

[12] Gibbon J (1977). Scalar expectancy theory and Weber’s law in animal timing. Psychological Review.

[13] Wearden JH, Pentonvoak IS (1995). Feeling the heat: Body temperature and the rate of subjective time, revisited. Q J Exp Psychol-B.

[14] Angrilli A, Cherubini P, Pavese A, Manfredini S (1997). The influence of affective factors on time perception. Percept Psychophys.

[15] Mella N, Conty L, Pouthas V (2011). The role of physiological arousal in time perception: Psychophysiological evidence from an emotion regulation paradigm. Brain Cognition.

[16] Droit-Volet S, Brunot S, Niedenthal PM (2004). Perception of the duration of emotional events. Cognition & emotion.

[17] Zhang X, Zhou XL (2007). Time perception of emotional events. Progress in Natural Science.

[18] Gil S, Droit-Volet S (2011). “Time flies in the presence of angry faces”… depending on the temporal task used!. Acta Psychol.

[19] Dirnberger G (2012). Give it time: Neural evidence for distorted time perception and enhanced memory encoding in emotional situations. Neuroimage.

[20] Tipples J (2010). Time flies when we read taboo words. Psychon B Rev.

[21] Gorn GJ, Chattopadhyay A, Sengupta J, Tripathi S (2004). Waiting for the Web: How screen color affects time perception. Journal of Marketing Research.

[22] Shibasaki, M. & Masataka, N. The color red distorts time perception for men, but not for women. *Scientific Reports* **4,** 5899 (2014). 10.1038/srep05899 PMC4116623 25077928

[23] Smets G (1969). Time expression of red and blue. Percept Motor Skill.

[24] Thönes S, Oberfeld D (2017). Meta-analysis of time perception and temporal processing in schizophrenia: Differential effects on precision and accuracy. Clinical Psychology Review.

[25] Antick JR, Schandler SL (1993). An exploration of the interaction between variation in wavelength and time perception. Percept Motor Skill.

[26] Katsuura T, Yasuda T, Shimomura Y, Iwanaga K (2007). Effects of monochromatic light on time sense for short intervals. Journal of Physiological Anthropology.

[27] Caldwell JA, Jones GE (1985). The effects of exposure to red and blue light on physiological indices and time estimation. Perception.

[28] Mikellides B (1990). Color and physiological arousal. Journal of Architectural and Planning Research.

[29] Grondin S, Laflamme V, Gontier E (2014). Effect on perceived duration and sensitivity to time when observing disgusted faces and disgusting mutilation pictures. Atten Percept Psycho.

[30] Huynh H, Feldt LS (1980). Performance of traditional F-tests in repeated measures designs under covariance heterogeneity. Communications in Statistics Part a-Theory and Methods.

[31] Cohen, J. *Statistical power analysis for the behavioral sciences*. 2nd edn, (L. Erlbaum Associates, 1988).

[32] Oberfeld D, Franke T (2013). Evaluating the robustness of repeated measures analyses: The case of small sample sizes and nonnormal data. Behavior Research Methods.

[33] Bartlett MS (1936). The square root transformation in analysis of variance. Supplement to the Journal of the Royal Statistical Society.

[34] Ulrich R, Vorberg D (2009). Estimating the difference limen in 2AFC tasks: Pitfalls and improved estimators. Attention, Perception & Psychophysics.

[35] Nachmias J (2006). The role of virtual standards in visual discrimination. Vision Research.

[36] Dyjas, O. & Ulrich, R. Effects of stimulus order on discrimination processes in comparative and equality judgements: Data and models. *The Quarterly Journal of Experimental Psychology*, 1–31 (2013). 10.1080/17470218.2013.847968 24295428

[37] Lang, P. J. In *Technology in Mental Health Care Delivery* Systems (eds J. B. Sidowski, J. H. Johnson, & T. A. Williams) 119–137 (Ablex, 1980).

[38] Lang PJ, Greenwald MK, Bradley MM, Hamm AO (1993). Looking at pictures: Affective, facial, visceral, and behavioral reactions. Psychophysiology.

[39] Provencio I (2000). A novel human opsin in the inner retina. Journal of Neuroscience.

[40] Brainard GC (2001). Action spectrum for melatonin regulation in humans: Evidence for a novel circadian photoreceptor. Journal of Neuroscience.

[41] Berson DM, Dunn FA, Takao M (2002). Phototransduction by retinal ganglion cells that set the circadian clock. Science.

[42] Moore RY (1983). Organization and function of a central nervous-system circadian oscillator - the suprachiasmatic hypothalamic nucleus. Fed Proc.

[43] Aston-Jones G (2005). Brain structures and receptors involved in alertness. Sleep Medicine.

[44] Thapan K, Arendt J, Skene DJ (2001). An action spectrum for melatonin suppression: evidence for a novel non-rod, non-cone photoreceptor system in humans. Journal of Physiology-London.

[45] Cohen RA, Barnes HJ, Jenkins M, Albers HE (1997). Disruption of short-duration timing associated with damage to the suprachiasmatic region of the hypothalamus. Neurology.

[46] Lewis PA, Miall RC (2009). The precision of temporal judgement: milliseconds, many minutes, and beyond. Philosophical Transactions of the Royal Society B-Biological Sciences.

[47] Dacey DM (2005). Melanopsin-expressing ganglion cells in primate retina signal colour and irradiance and project to the LGN. Nature.

[48] Ishihara, S. *The Series of Plates Designed as a Test of Colour Deficiency. 24 Plates Edition*., (Kanehara Trading Inc., 2013).

[49] Commission Internationale de l'Éclairage. Vol. CIE S 014-1/E:2006/ISO 11664-1:2008(E) (2006).

[50] Commission Internationale de l'Éclairage. Vol. CIE S 014-4/E:2007 (ISO 11664-4:2008) (2007).

[51] Lübbe, E. *Farbempfindung, Farbbeschreibung und Farbmessung. Eine Formel für die Farbsättigung*. (Springer Vieweg, 2013).

