# Supplementary material for: Color and time perception: Evidence for temporal overestimation of blue stimuli
Source: Sci Rep. 2018 Jan 26;8:1688. doi: 10.1038/s41598-018-19892-z (PMC5786107; doi:10.1038/s41598-018-19892-z)
Supplement: Supplementary file 1 — Supplementary Information [file 41598_2018_19892_MOESM1_ESM.pdf]

Supplementary Information to

Color and time perception: Evidence for temporal overestimation of blue stimuli

Thönes, S., von Castell, C., Iflinger, J., and Oberfeld, D.

Durations difference limens as a function of comparison position

In two-interval duration discrimination tasks, the duration difference limens (DLs) were reported to depend on the presentation order of standard and comparison [1,2]. Typically, smaller DLs are observed when the comparison is presented in the second interval (stimulus order <*sc*>, where *c* denotes the comparison and *s* denotes the standard) rather than in the first interval (stimulus order <*cs*>). For this reason, in addition to the main analyses, we fitted a separate cumulative-normal psychometric function (PMF) to the observed responses, for each ***position of the comparison interval*** (CI position).

The mean DLs for each color sequence (b-r, r-b, b-b, r-r) and each **CI position (first position: <cs>, second position: <sc>**) are presented in Supplementary Table 1.

The data were analyzed by means of an rmANOVA including the within-subjects factors color sequence (r-b, b-r, b-b, r-r) and CI position (<cs>, <sc>). There was no significant effect of color sequence on the DL, $F(3, 36) = 1.306$, $\tilde{\varepsilon} = .788$, $p = .290$, $\eta_p^2 = .098$, supporting the results of the main analysis. The color sequence x CI position interaction was also not significant, $F(3, 36) = 0.848$, $\tilde{\varepsilon} = .768$, $p = .453$, $\eta_p^2 = .066$. However, there was significant effect of CI position on the DL, $F(1, 12) = 7.428$, $p = .018$, $\eta_p^2 = .382$. The DLs were higher (i.e., the duration judgments were less precise) on trials where the CI was presented first. This result is compatible with previous studies on duration discrimination [1-3].

References

1 Dyjas, O. & Ulrich, R. Effects of stimulus order on discrimination processes in comparative and equality judgements: Data and models. *The Quarterly Journal of Experimental Psychology*, 1-31, doi:10.1080/17470218.2013.847968 (2013).

2 Ulrich, R. & Vorberg, D. Estimating the difference limen in 2AFC tasks: Pitfalls and improved estimators. *Attention, Perception, & Psychophysics* **71**, 1219-1227, doi:10.3758/App.71.6.1219 (2009).

3 Nachmias, J. The role of virtual standards in visual discrimination. *Vision Research* **46**, 2456-2464, doi:10.1016/j.visres.2006.01.029 (2006).

Supplementary Table 1

Means and standard deviations (in parentheses) of the estimated DLs, as a function of color sequence and CI position.

**CI position**	Color Sequence	DL [ms]
**first (<cs>)**	**mean**	**141.79 (54.65)**
	b-r	129.68 (66.68)
	r-b	152.57 (77.70)
	b-b	135.28 (65.17)
	r-r	149.62 (47.35)
**second (<sc>)**	**mean**	**111.58 (52.29)**
	b-r	108.77 (50.88)
	r-b	98.38 (32.02)
	b-b	114.25 (50.27)
	r-r	124.91 (95.69)
